# First person – José Britto-Júnior

**DOI:** 10.1242/bio.058305

**Published:** 2021-01-20

## Abstract

First Person is a series of interviews with the first authors of a selection of papers published in Biology Open, helping early-career researchers promote themselves alongside their papers. José Britto-Júnior is first author on ‘The basal release of endothelium-derived catecholamines regulates the contractions of Chelonoidis carbonaria aorta caused by electrical-field stimulation’, published in BiO. José conducted the research described in this article while a master's student in Professor Matheus L. Rocha's laboratory at Faculty of Pharmacy, University of Goias, and is now a PhD student in the Department of Pharmacology at the University of Campinas, Brasil, investigating basic cardiovascular pharmacology, endothelium, endothelial catecholamines and comparative physiology.


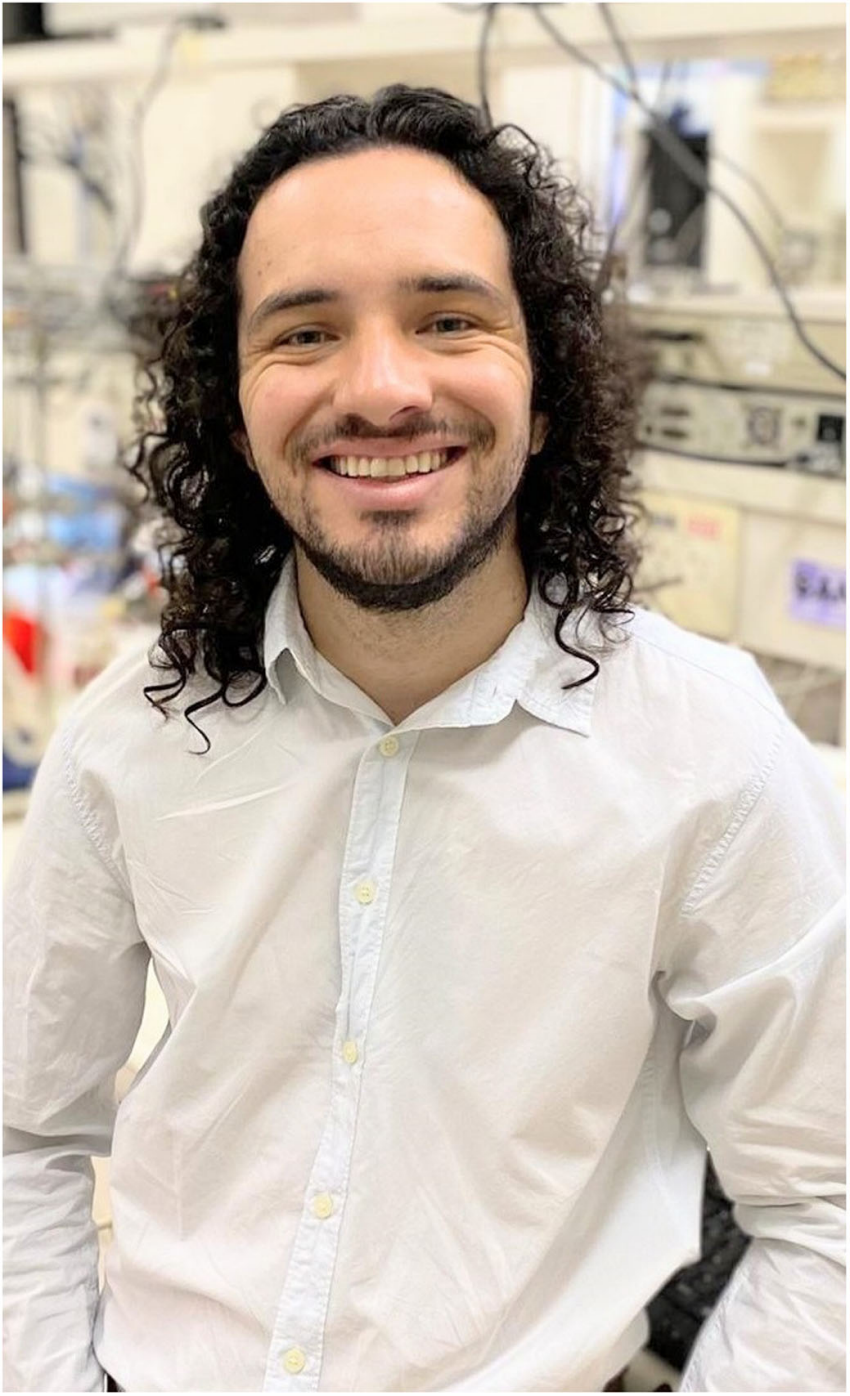


**José Britto-Júnior**

**What is your scientific background and the general focus of your lab?**

Basic vascular pharmacology. The lab is focused on endothelium-derived catecholamines.

**How would you explain the main findings of your paper to non-scientific family and friends?**

We have demonstrated that tortoise vascular tissue presents basal release of catecholamines. These substances are important vasoactive mediators and until now thought to be released only from nerve terminals and adrenal glands.

“We have demonstrated that tortoise vascular tissue presents basal release of catecholamines.”

**What are the potential implications of these results for your field of research?**

These endothelium-derived mediators may constitute important therapeutic targets for treatment of vascular diseases such as hypertension.

**What has surprised you the most while conducting your research?**

In this era of molecular biology, how interesting are bioassay experiments.

**Figure:**
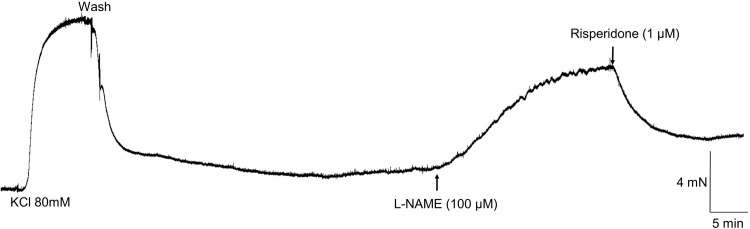
**Representative tracing showing the reversal by risperidone (1 µM; n = 5/5) of the elevated tonus induced by L-NAME (100 µM) in aortic rings of *Chelonoidis carbonaria*.**

**What, in your opinion, are some of the greatest achievements in your field and how has this influenced your research?**

The discovery of nitric oxide and endothelin release by endothelial cells.

**What changes do you think could improve the professional lives of early-career scientists?**

Increase availability of fellowships for post-graduate education.

**What's next for you?**

To do a post-doc after finishing my PhD.
